# Genome-Wide Identification and Expression Analyses of *AnSnRK2* Gene Family under Osmotic Stress in *Ammopiptanthus nanus*

**DOI:** 10.3390/plants10050882

**Published:** 2021-04-27

**Authors:** Yueming Tang, Fengzhong Lu, Wenqi Feng, Yuan Liu, Yang Cao, Wanchen Li, Fengling Fu, Haoqiang Yu

**Affiliations:** Key Laboratory of Biology and Genetic Improvement of Maize in Southwest Region, Maize Research Institute, Sichuan Agricultural University, Chengdu 611130, China; tangyueming@stu.sicau.edu.cn (Y.T.); lufz@stu.sicau.edu.cn (F.L.); fwq@stu.sicau.edu.cn (W.F.); 18394159617@163.com (Y.L.); caoy@stu.sicau.edu.cn (Y.C.); aumdyms@sicau.edu.cn (W.L.)

**Keywords:** protein kinase, SnRK2, osmotic stress, expression profiles, *Ammopiptanthus nanus*

## Abstract

Sucrose non-fermenting-1 (SNF1)-related protein kinase 2’s (SnRK2s) are plant-specific serine/threonine protein kinases and play crucial roles in the abscisic acid signaling pathway and abiotic stress response. *Ammopiptanthus nanus* is a relict xerophyte shrub and extremely tolerant of abiotic stresses. Therefore, we performed genome-wide identification of the *AnSnRK2* genes and analyzed their expression profiles under osmotic stresses including drought and salinity. A total of 11 *AnSnRK2* genes (*AnSnRK2.1-AnSnRK2.11*) were identified in the *A. nanus* genome and were divided into three groups according to the phylogenetic tree. The *AnSnRK2.6* has seven introns and others have eight introns. All of the AnSnRK2 proteins are highly conserved at the N-terminus and contain similar motif composition. The result of *cis*-acting element analysis showed that there were abundant hormone- and stress-related *cis*-elements in the promoter regions of *AnSnRK2s*. Moreover, the results of quantitative real-time PCR exhibited that the expression of most *AnSnRK2*s was induced by NaCl and PEG-6000 treatments, but the expression of *AnSnRK2.3* and *AnSnRK2.6* was inhibited, suggesting that the *AnSnRK2s* might play key roles in stress tolerance. The study provides insights into understanding the function of *AnSnRK2s*.

## 1. Introduction

Plants frequently encounter surrounding environment changes and are vulnerable to these stimuli, including drought, extreme temperatures, and salinity. After long-term evolution, plants have evolved a variety of mechanisms to adapt to abiotic stresses. Protein phosphorylation or dephosphorylation is one of the most important cures for plant response to environmental stresses [1,2,3]. Protein phosphorylation catalyzed by protein kinases interconnects various signal pathways and plays a vital role in plant response to abiotic stresses [4]. For instance, the plant mitogen-activated protein kinase (MAPK) mediates MAPKKK–MAPKK–MAPK cascade reaction through phosphorylation to respond to a variety of biotic and abiotic stresses [5,6]. Phosphorylated AtMKKK1 interacts with AtMKK1 and AtMKK2 to activate MPK4 in drought response [7]. Calcium-dependent protein kinase (CDPK) can phosphorylate and activate transcription factors, thereby regulating gene expression in response to environmental stimuli [8,9]. AtCPK4 and AtCPK11 phosphorylate two ABA-responsive transcription factors (ABF1 and ABF4) in response to abscisic acid (ABA) and drought stress [10]. The sucrose non-fermenting-1 (SNF1)-related protein kinases (SnRKs) are plant-specific serine/threonine protein kinases. They widely exist in higher plants, where they regulate plant growth and development and response to adversity stresses [11,12,13,14,15]. According to the sequence similarity and conservation of the C-terminal domain, the SnRK proteins are classified into three subfamilies, namely SnRK1, SnRK2, and SnRK3 [16,17]. The SnRK1 subfamily has been reported to participate in sugar and ABA signaling pathways and metabolic regulation [18,19,20]. SnRK2 and SnRK3 subfamilies mediate plant response to drought, salinity, and osmotic stress [21,22].

Notably, SnRK2s control plant growth, development, and stress response via the ABA signaling pathway [23]. In plants, *SnRK2s* were first identified by the description of the *PKABA1* gene in wheat, which is involved in ABA signal transduction. The expression of *PKABA1* is induced by dehydration, salinity, low-temperature, and osmotic stress [24]. Likewise, PKABA1 phosphorylates the ABA response element-binding factor (TaABF) to regulate grain maturation and seed dormancy [25,26]. Furthermore, the expression of the *SnRK2* genes was induced by osmotic stress of mannitol and NaCl, or ABA, in *Arabidopsis thaliana* [27]. The SnRK2.2/2.3/2.6 phosphorylate ABA-responsive element-binding protein (AREB) and thus positively regulate ABA signaling in response to drought stress in *Arabidopsis* [28]. In addition, SnRK2s regulate root morphogenesis, flowering, fruit maturation, yield formation, and plant height [15,29,30,31,32]. Under saline conditions, the *AtSnRK2.4/2.10* knockout mutant shows reduced root length and fewer lateral roots than the wild type [33]. Overexpression of the *TaSnRK2.9-5A* gene increases the grain yield in transgenic rice [31].

*A. nanus* is a relict broadleaf shrub. It has survived in the desert and arid regions of Central Asia since the disappearance of the ancient Mediterranean in the tertiary period [34]. It is extremely tolerant to abiotic stresses such as drought, salinity, barrenness, and extreme temperatures [35,36]. Considering the crucial roles of SnRK2s in stress response, we analyzed and identified the *SnRK2* genes in the genome of *A. nanus* through bioinformatics. Subsequently, quantitative real-time PCR was performed to analyze the expression pattern of the *AnSnRK2* genes under osmotic stresses including drought and salinity. The study will provide useful information for further functional studies of the *AnSnRK2* genes.

## 2. Results

### 2.1. The SnRK2 Genes in A. nanus

The amino acid sequences of SnRK2s from *Arabidopsis* and rice were used to perform local blast in *A. nanus*. In total, 224 candidate sequences were obtained. After removing the redundancy, 215 candidate sequences were aligned with the SnRK2 sequences of *Arabidopsis* and rice to construct a phylogenetic tree. The results showed that 11 SnRK2s of *A. nanus* and all SnRK2s of *Arabidopsis* and rice were clustered into the same branch with a bootstrap value of 100 (Figures S1 and S2). The 11 *AnSnRK2s* candidate genes were named *AnSnRK2.1*–*AnSnRK2.11*. Their coding sequences were 1017 to 1152 bp in length, encoding 338 to 383 amino acids, with a molecular weight of 38.42 to 43.07 KDa, respectively. The theoretical isoelectric point of AnSnRK2 proteins ranged from 4.71 to 6.21, indicating that they were acidic proteins. Ten AnSnRK2s were predicted to be hydrophilic proteins with grand average of hydropathicity (GRAVY) < 0. No signal peptides were detected, and only AnSnRK2.1 showed a transmembrane domain (Figure S3). Prediction of subcellular location indicated that AnSnRK2s localized in cytoskeleton and cytosol, which was consistent with their hydrophilic nature. Among them, only AnSnRK2.1 localized in chloroplast, which might be involved in the photosynthetic metabolism of cells (Table 1).

### 2.2. Multiple Alignment and Phylogenetic Analysis of AnSnRK2s

**Figure 1:**
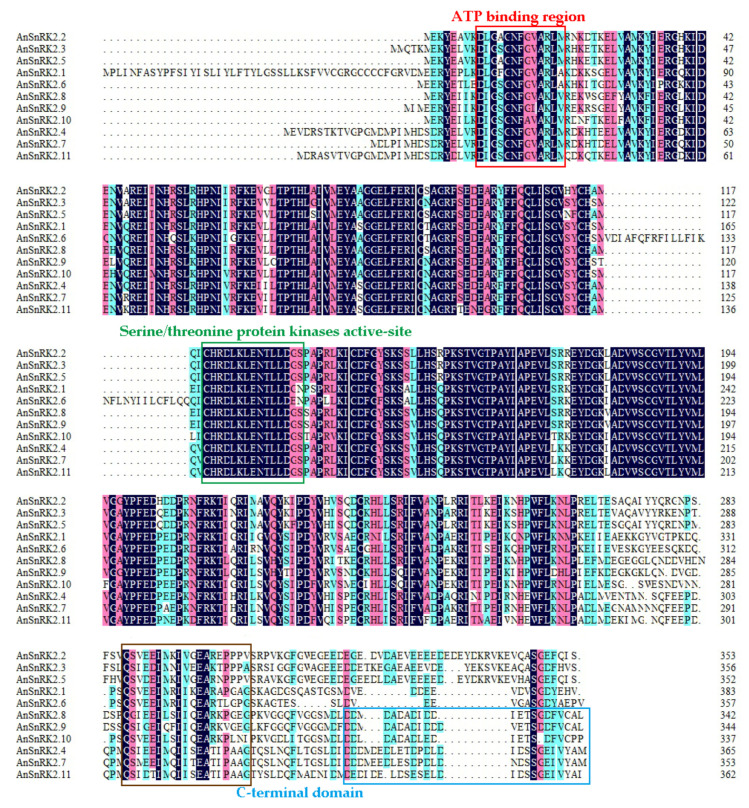
Multiple alignment of amino acid sequences of AnSnRK2s. The ATP-binding domain and the serine/threonine protein kinase active-site are marked by red and green boxes, respectively. The C-terminal domain contains two subdomains. The brown box represents the structural domain response to abiotic stress; the blue box represents ABA inducible regulatory domain.

The results of multiple alignment indicated that 11 AnSnRK2s were highly conserved with an average of 66.7% sequence identity. Specifically, all AnSnRK2s possessed a highly conserved protein kinase domain in the N-terminal, including an ATP-binding region, the serine/threonine protein kinase active-site, and an abiotic stress activation region in C-terminal. AnSnRK2.4/7/8/9/10/11 contained abundant aspartic acids (D) and glutamic acids (E) at the C-terminus, which were proved to regulate ABA signaling activity (Figure 1) [37,38].

**Figure 2:**
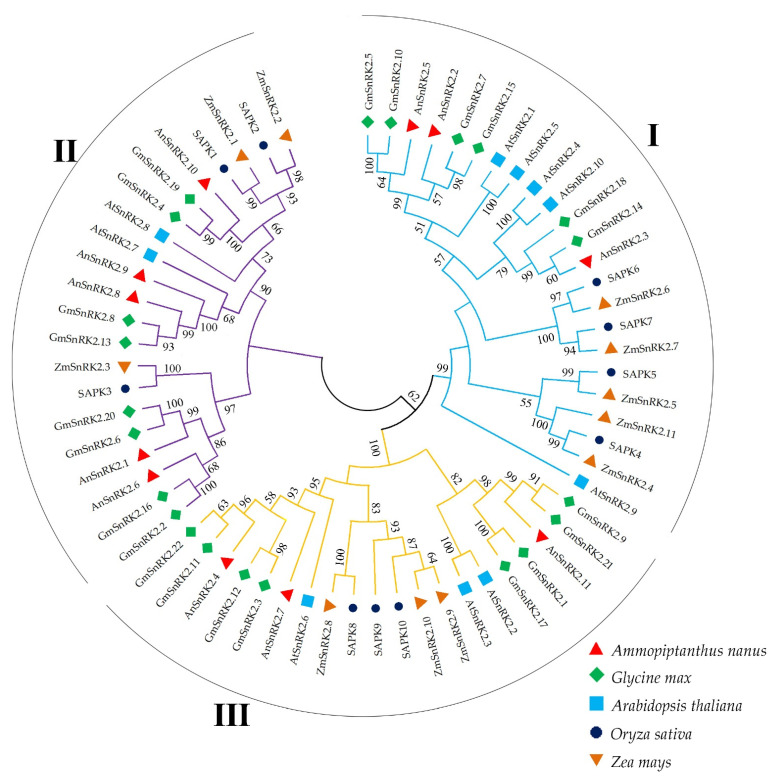
Phylogenetic tree of SnRK2s from *Arabidopsis* (*Arabidopsis thaliana*), rice (*Oryza sativa* Japonica), maize (*Zea mays*), soybean (*Glycine max*), and *A. nanus*. Members in the same subclade share a unique color. I, II, and III represent three subclades.

Furthermore, the SnRK2 amino acid sequences of *A. nanus*, *Arabidopsis*, rice, maize, and soybean were used to construct a phylogenetic tree. As shown in Figure 2, these SnRK2s were divided into three subgroups (I, Ⅱ, and Ⅲ), which was consistent with previous classifications [27,38]. The AnSnRK2s showed a closer phylogenetic relationship with GmSnRK2s of soybean than the SnRK2s of *Arabidopsis*, rice, and maize. The AnSnRK2.2, AnSnRK2.3, and AnSnRK2.5 were classed into one group (Subgroup I) and showed a diversity at C-terminal compared to other AnSnRK2s (Figure 1). The AnSnRK2.1, AnSnRK2.6, AnSnRK2.8, AnSnRK2.9, and AnSnRK2.10 were divided into one subgroup (Subgroup II). The AnSnRK2.4, AnSnRK2.7, and AnSnRK2.11 were clustered into the same subgroup (Subgroup III) and exhibited high conservation at C-terminal. The result indicates that the SnRK2 genes are relatively conservative in evolution.

### 2.3. Gene Structure and Motifs of AnSnRK2s

**Figure 3:**
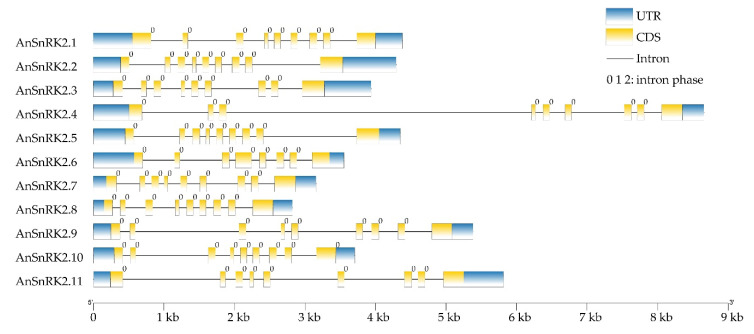
The exon–intron organizations of *AnSnRK2s.*

As shown in Figure 3, gene structure analysis showed that all *AnSnRK2s* had un-translation regions (UTRs) at both 5 and 3 terminals. Only the *AnSnRK2.6* gene had eight exons; other *AnSnRK2s* possessed nine exons with different lengths. The lengths of the first and last exon of every *AnSnRK2* were different. The lengths of the second to the seventh exons of *AnSnRK2.6* were 75, 102, 231, 93, 105, and 99 bp, respectively. However, the length of the second to the eighth exons of the other *AnSnRK2*s were 75, 102, 54, 93, 93, 105, and 99 bp, respectively. All introns of the *AnSnRK2* genes were 0-phase, which interrupted the exons between two triplet codons, showing high conservation of exon–intron structure and similar splicing patterns of *AnSnRK2s* [39].

**Figure 4:**
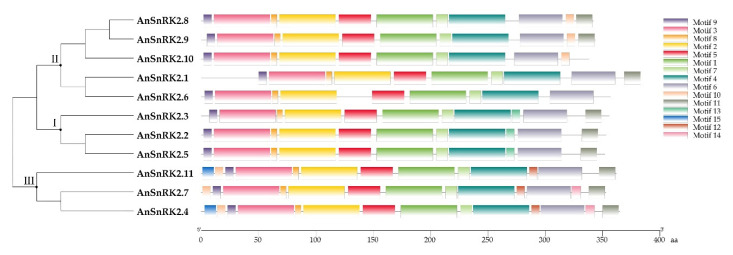
The motif composition of AnSnRK2s.

To further understand the systematic relationship among AnSnRK2 proteins, 15 conserved motifs of AnSnRK2s were predicted using MEME (Figure 4). All AnSnRK2s possessed nine conserved motifs and exhibited similar motif composition within subgroups. The motif 13 was only shared by Subgroup I AnSnRK2s, which was different from other subgroups. The motifs 12, 14, and 15 were only shared by subgroup III AnSnRK2s. Among them, the AnSnRK2s of subgroups II and III showed motif diversity at the C-terminal.

### 2.4. The cis-Elements in the Promoter of AnSnRK2s

**Figure 5:**
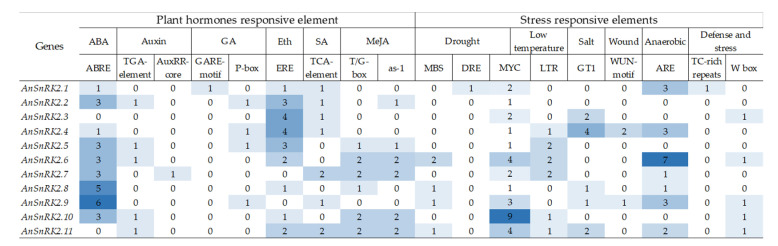
The *cis*-elements in *AnSnRK2* promoters. The number represents the number of *cis*-elements.

Through PlantCARE analysis, a total of 75 *cis*-elements were found in the promoter region of the *AnSnRK2* genes, including 9 types of hormone-related elements (responding to ABA, auxin, gibberellin, ethylene, salicylic acid, and methyl jasmonate), 9 types of stress-related response elements (response to drought, salt, low-temperature, wound, anaerobic, defense, and stress responses), and 13 types of light-related response elements (Figure 5). Notably, ABA-responsive elements (ABREs) were found in the promoters of most *AnSnRK2s* except *AnSnRK2.3* and *AnSnRK2.11*, indicating their potential roles in ABA signaling. Meanwhile, ethylene-responsive elements (EREs) were found in the promoters of *AnSnRK2s* except for *AnSnRK2.7* and *AnSnRK2.9*. MYB binding sites (MBSs), MYC-binding sites, and low-temperature inducing elements (LTRs) were also found in the promoters of most *AnSnRK2s*. The results suggest that *AnSnRK2s* not only respond to ABA but also are involved in stress response.

### 2.5. Expression of AnSnRK2 Genes under Drought and Salinity Stress

**Figure 6:**
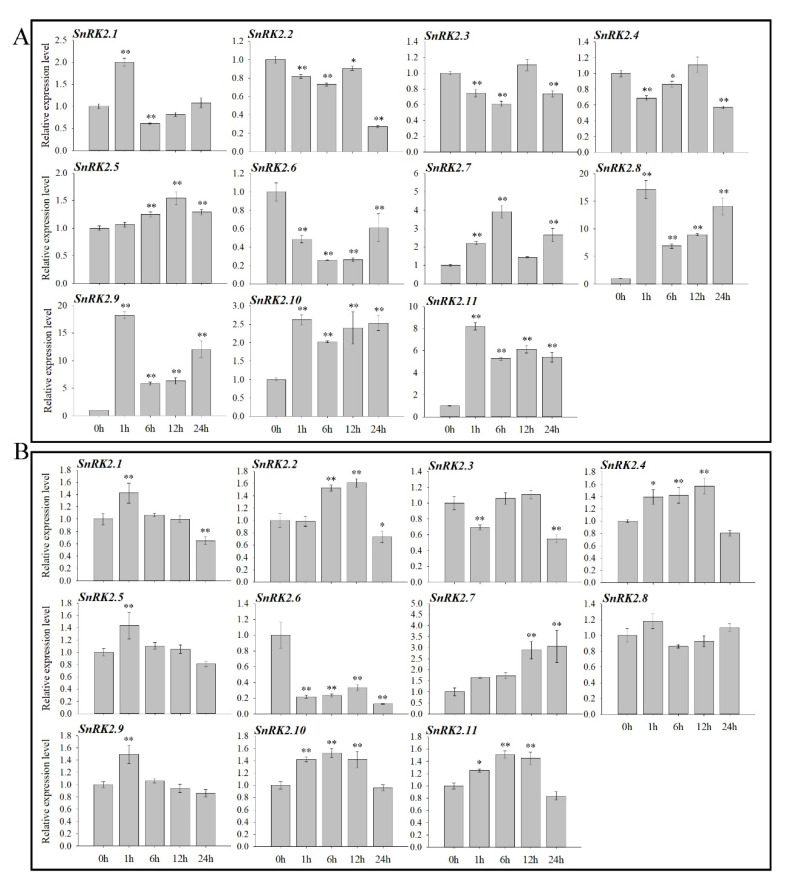
Expression profiles of *AnSnRK2s* under salinity and drought stresses. *A. nanus* plants were subjected to 250 mM NaCl (**A**) and 20% PEG-6000 treatments (**B**).Three independent experiments were performed and error bars indicate standard deviation (Student’s *t*-test; * *p* < 0.05; ** *p* < 0.01).

To investigate the expression of the *AnSnRK2* genes in abiotic stress response, their expression patterns under the high osmotic pressure (PEG-6000 or NaCl) were analyzed by qRT-PCR. The results of qRT-PCR showed that the expression of *AnSnRK2.1*, *AnSnRK2.5*, *AnSnRK2.7*, *AnSnRK2.8*, *AnSnRK2.9*, *AnSnRK2.10*, and *AnSnRK2.11* was significantly upregulated by salt treatment (250 mM NaCl) and peaked at 1, 12, 6, 1, 1, 1, and 1 h of treatment, respectively. The expression of *AnSnRK2.8*, *AnSnRK2.9*, and *AnSnRK2.11* was upregulated more than 17-, 18-, and 8-fold, respectively. However, the expression of *AnSnRK2.2 AnSnRK2.3 AnSnRK2.4*, and *AnSnRK2.6* was inhibited by salt and reached a minimum at 24, 6, 1, and 6 h of treatment, respectively (Figure 6A). After 20% PEG-6000 treatment, the expression of *AnSnRK2.3* and *AnSnRK2.6* was significantly downregulated and decreased to 55% and 13% at 24 h, respectively, compared to control (0 h). The expression of *AnSnRK2.6* was significantly inhibited by both treatments and 33% lower than control. The *AnSnRK2.8* exhibited no differential expression. Only *AnSnRK2.7* was continuously induced during stress treatment. The results indicate that the *AnSnRK2s* may play crucial roles in osmotic stress response.

## 3. Discussion

SnRK2s are plant-specific Ser/Thr protein kinases and play crucial roles in plant growth and stress response [40]. To date, the *SnRK2* genes have been identified from different species, such as rice (10), maize (10), sorghum (10), pak choi (13), Chinese white pear (10), and pepper (9) [17,41,42,43,44,45]. It has been shown that the number of *SnRK2* genes ranges from 8 (*Solanum tuberosum*) to 22 (*Glycine max*) [46,47]. However, most of them have 9–11 *SnRK2s*. In the case of soybeans, it may be due to two whole-genome duplication events [48]. In this study, 11 *AnSnRK2*s were identified from the *A. nanus* (Table 1). 

The phylogenetic tree showed that AnSnRK2s were divided into three groups (Figure 2). The AnSnRK2s from the same subgroup showed similar conserved domains, motif composition, and gene architecture and slight diversity among different subgroups (Figure 1, Figure 3, and Figure 4). Most of *SnRK2* genes identified from different species have eight introns, but few of them have one (*ZmSnRK2.5*), two (*SbSnRK2.8*), three (*SAPK5*), five (*AtSnRK2.8*), six (*SAPK10*/*StSnRK2.6*), seven (*GmSnRK2.6*/*SbSnRK2.2*/*ZmSnRK2.9*/*ZmSnRK2.10*), or nine (*AtSnRK2.6*/*SbSnRK2.7*) introns [17,27,41,42,46,47]. It is speculated that the number of introns of the plant *SnRK2* gene is highly conservative at eight. In this study, the *AnSnRK2* genes all had eight introns except *AnSnRK2.6* (Figure 3). The lengths of the second to eighth exons of all *SnRK2s* from maize and *Arabidopsis* are 75, 102, 54, 93, 93, 105, and 99 bp, respectively [41], which is likewise found in *A. nanus*. The *AnSnRK2.6* gene only possessed seven introns because of an extended fourth exon. These results indicate *SnRK2s* evolve structure conservation among the same subclades and diversity between different subgroups.

When plants are exposed to stress, the stress-related transcription factors will be activated through a series of signal transmissions. These activated factors will combine with the *cis*-acting elements of downstream target gene promoters and regulate their expression to respond to stress [49]. The study of *cis*-acting elements of genes is particularly important to reveal their potential roles. In this study, the *AnSnRK2s* promoters possessed abundant hormone- and stress-responsive *cis*-elements. The MYC, ABRE, or ERE *cis*-elements were enriched in most of the *AnSnRK2s* promoters, which are also found in pepper and cotton [45,50]. Meanwhile, a huge number of cold-inducing elements (LTRs) were identified in *AnSnRK2s* promoters (Figure 5). The result suggests that *AnSnRK2s* play an important role in the ABA signaling pathway and stress response.

It has been proved that *SnRK2* is involved in abiotic stresses and used to improve plants’ stress resistance via the expression of *SnRK2* genes. The expression of *TaSnRK2.4* in wheat, *SAPK4* in rice, *NtSnRK2.2* in tobacco, and *MpSnRK2.10* in apple can be activated by salt, water deficit, low temperature, or oxidative stresses. Overexpression of *TaSnRK2.4*, *SAPK4*, *NtSnRK2.2*, or *MpSnRK2.10* increases the tolerance to salt, drought, cold, or oxidative stress of transgenic plants, respectively [12,14,51,52]. In this study, all *AnSnRK2*s were found to respond to osmotic stress except for *SnRK2.8*. Only *AnAnRK2.7* can be upregulated by both NaCl and PEG, while *AnSnRK2.6* is inhibited by these stresses (Figure 6). It is speculated that these two genes may play an extraordinary role under osmotic stress.

In summary, we identified 11 *AnSnRK2* members from *A. nanus* and analyzed their gene structures, conservative motifs, phylogenetic relationships, *cis*-acting elements, and expression profiles under osmotic stress. The results provide valuable information for further elucidating the function of *AnSnRK2s*.

## 4. Materials and Methods 

### 4.1. Identification of AnSnRK2 Genes in A. nanus 

In order to identify the *AnSnRK2* gene of *A. nanus*, the protein and genome sequences were obtained from *A. nanus* genome project [53]. The 10 SnRK2 protein sequences from Arabidopsis and rice were retrieved from the Arabidopsis Information Resource (http://www.arabidopsis.org/, accessed on 15 October 2020) and Rice Genome Annotation Project (http://rice.plantbiology.msu.edu/index.shtml, accessed on 15 October 2020), respectively [17,27], and used as queries to perform local BLASTP (blast-2.9.0) with an E-value 1 × 10^10^ and homology minimum 50% to obtain the SnRK2 of *A. nanus*. The amino acid sequences of candidates were analyzed by using the Hidden Markov Model (HMM) seed profiles of the protein kinase domain (PF00069) and protein serine/threonine kinase (PF07714) from the Pfam database (http://pfam.xfam.org/, accessed on 15 October 2020). After removing the redundancy sequences, the candidate genes were obtained and used to construct a phylogenetic tree with SnRK2s of the *Arabidopsis* and rice. The candidate genes clustered with *Arabidopsis* and rice SnRK2s were identified as putative *AnSnRK2s*. The motif composition of AnSnRK2s was analyzed using the online tool MEME V4.12.0 (http://meme.sdsc.edu/meme/meme.html, accessed on 20 October 2020) with the motif length set at 10–100 and motif maximum number set at 15. The protein molecular weight, isoelectric point, and protein hydrophobicity of AnSnRK2s were predicted using online ProtParam software provided by ExPaSy (available online: http://expasy.org/tools/protparam.html, accessed on 20 October 2020). The subcellular location and transmembrane structure of the AnSnRK2s were predicted by the WoLF PSORT tools (https://wolfpsort.hgc.jp/, accessed on 20 October 2020) and the TMHMM v.2.0 (http://www.cbs.dtu.dk/services/TMHMM-2.0/, accessed on 20 October 2020), respectively.

### 4.2. Multiple Alignment and Phylogenetic Analysis

DNAMAN (version 8) software was used for the multiple alignment of the amino acid sequences of AnSnRK2s. The conserved domains were analyzed using the Conserved Domain Search Database (CDD, http://www.ncbi.nlm.nih.gov/Structure/cdd/cdd.shtml, accessed on 15 October 2020). Subsequently, the phylogenetic tree of SnRK2s between *A. nanus*, *Arabidopsis*, rice, maize, and soybean was constructed using MEGA X (version X, Hachioji, Tokyo, Japan) with neighbor-joining method and 1000 bootstrap replicates. The SnRK2 gene IDs of *Arabidopsis*, rice, maize, and soybean are listed in Table S1.

### 4.3. Gene Structure and Analysis of cis-Acting Elements

The encoding and genomic sequences of *AnSnRK2s* were used to analyze exon–intron organizations and intron type by using Gene Structure Display Server (GSDS) (http://gsds.cbi.pku.edu.cn/index.php, accessed on 20 October 2020).

In order to analyze the *cis*-acting elements of *AnSnRK2* family, the 2000 bp region upstream of the start codon was obtained and used to analyze the *cis*-acting elements using PlantCARE online software (available online: http://bioinformatics.psb.ugent.be/webtools/plantcare/html/, accessed on 25 October 2020).

### 4.4. Plant Materials and Treatments

As described by Ding [54] with minor modification, the seeds of *A. nanus* collected from Tarim Basin in China were surface-sterilized with 75% (*v*/*v*) ethanol, immersed in sterilized water for swelling 24 h at 25 °C, sown in pots filled with nutrient soil (nutrient soil/vermiculite = 3:1), and cultured in a growth chamber under a photoperiod of 14 h light at 30 °C/10 h dark at 22 °C with 60–70% relative humidity. The five-leaf-old seedlings with the same size were treated with 20% PEG-6000 or 250 mM NaCl, with three replicates, as described by Yu et al. [55,56]. At 0 (control), 3, 6, 12 and 24 h of the treatments, the shoots from six seedlings were collected, ground in liquid nitrogen, and used for RNA extraction. Total RNA was extracted using RNAiso Plus kit and reversely transcribed into cDNA by using PrimeScript RT reagent Kit with gDNA Eraser (TAKARA, Dalian, China). The cDNA samples were stored at −20 °C.

### 4.5. qRT-PCR Analysis

A set of specific primers of *AnSnRK2s* and a pair of specific primers of *AnActin* (GenBank accession number: KJ873129) for the internal control were designed by Primer5.0 and synthesized at Sangon (China) (Table S2). The qRT-PCR was performed using SYBR Green Ⅰ kit (TAKARA, Dalian) in CFX-96 system (Bio-Rad, Hercules, CA, USA) as described by Yu et al. [57]. The 2^–ΔΔCT^ method of the CFX Manager software version 2.0 (Bio-Rad, USA) was used to normalize the expression differentiation between the internal control and the *AnSnRK2s* [58]. The data are presented as the mean values ± standard deviation (SD). The statistical significance among three biological replicates was tested by Microsoft Excel 2017 and SPSS 17.0 software based on Student’s *t*-tests.

## Figures and Tables

**Table 1:** Characteristics of putative *AnSnRK2* genes in *A. nanus.*

Gene Name	Gene ID	CDs(bp)	Amino Acid	Isoelectric Point	Molecular Weight (KDa)	Introns	Grand Average Hydropathy	Subcellular Localization (Probability)
*AnSnRK2.1*	EVM0003959.1	1152	383	5.92	43.07	8	−0.25	chloroplast
*AnSnRK2.2*	EVM0011565.1	1062	353	5.81	40.52	8	−0.53	cytoskeleton
*AnSnRK2.3*	EVM0013530.1	1071	356	6.21	40.71	8	−0.47	cytoskeleton
*AnSnRK2.4*	EVM0016282.1	1098	365	4.89	41.55	8	−0.34	cytoskeleton
*AnSnRK2.5*	EVM0017722.1	1059	352	5.95	40.60	8	−0.50	cytoskeleton
*AnSnRK2.6*	EVM0020312.1	1074	357	5.69	40.43	7	−0.19	cytoplasm
*AnSnRK2.7*	EVM0025729.1	1062	353	5.00	40.22	8	−0.35	cytoskeleton
*AnSnRK2.8*	EVM0028012.1	1029	342	5.24	38.76	8	−0.31	cytoplasm
*AnSnRK2.9*	EVM0029135.1	1035	344	5.23	38.96	8	−0.3	cytoskeleton
*AnSnRK2.10*	EVM0032503.1	1017	338	5.3	38.42	8	−0.2	cytoplasm
*AnSnRK2.11*	EVM0034033.1	1089	362	4.71	41.16	8	−0.27	cytoskeleton

## Supplementary Materials

The following are available online at https://www.mdpi.com/article/10.3390/plants10050882/s1, Figure S1: The phylogenetic tree constructed using 215 candidate sequences from *A. nanus* and SnRK2s of *Arabidopsis* and rice to identify AnSnRK2s. Figure S2: CDD results of 11 candidate members and EVM0005794 and EVM0001332. Figure S3: The trans-membrane structure prediction of AnSnRK2.1. Table S1: SnRK2 genes in *Arabidopsis*, rice, maize, and soybean. Table S2: The primers for qRT-PCR.
