# Supplementary material for: Genome-Wide Identification and Expression Analyses of AnSnRK2 Gene Family under Osmotic Stress in Ammopiptanthus nanus
Source: Plants (Basel). 2021 Apr 27;10(5):882. doi: 10.3390/plants10050882 (PMC8145913; doi:10.3390/plants10050882)
Supplement: Supplementary file 1 [file plants-10-00882-s001.zip › plants-1140913-supplementary.pdf]

Figure. S1 The phylogenetic tree constructed using 215 candidate sequences from *A. nanus* and SnRK2s of *Arabidopsis* and rice.

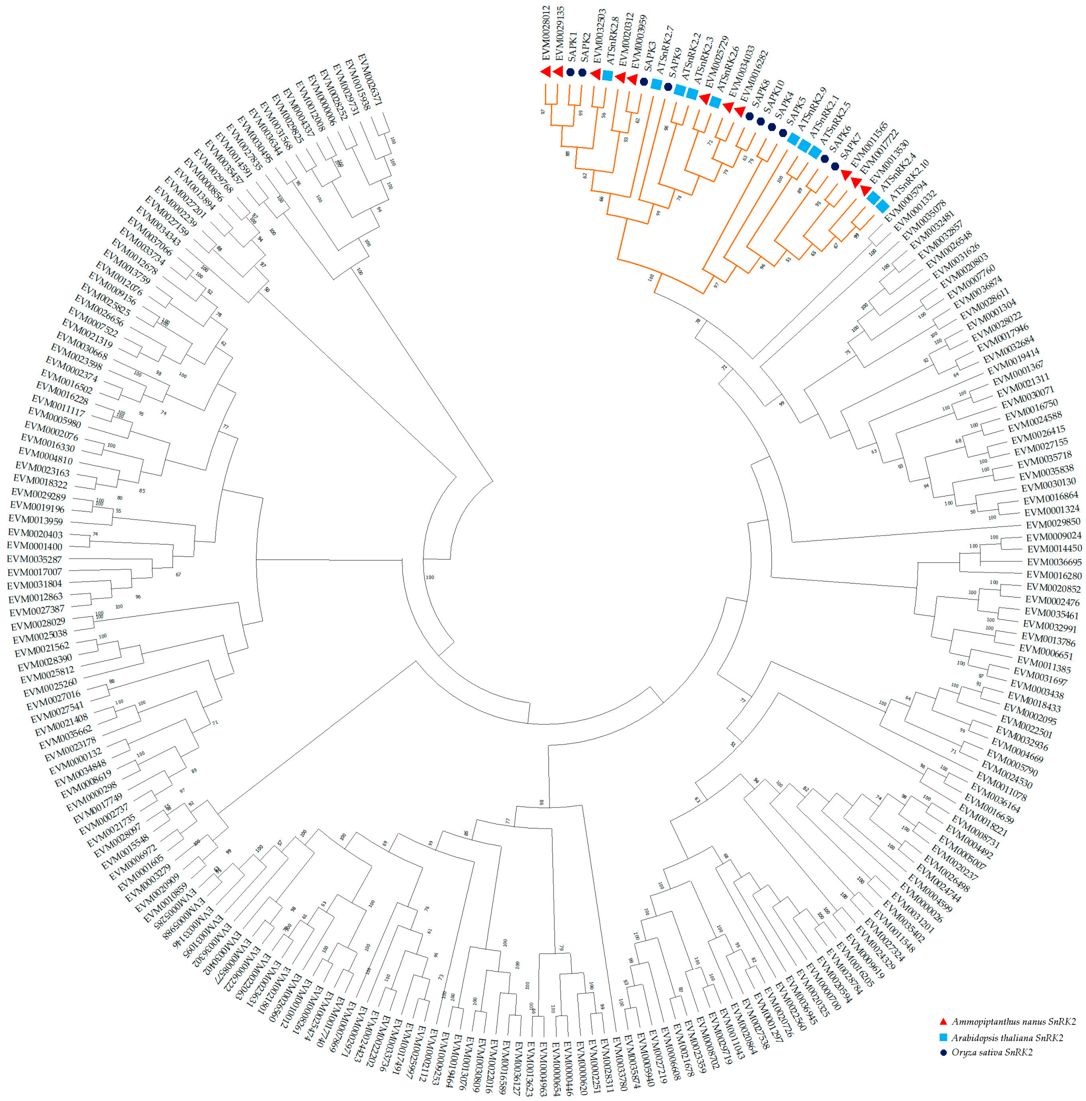

Figure. S2 CDD results of 11 candidate members and EVM0005794 and EVM0001332.

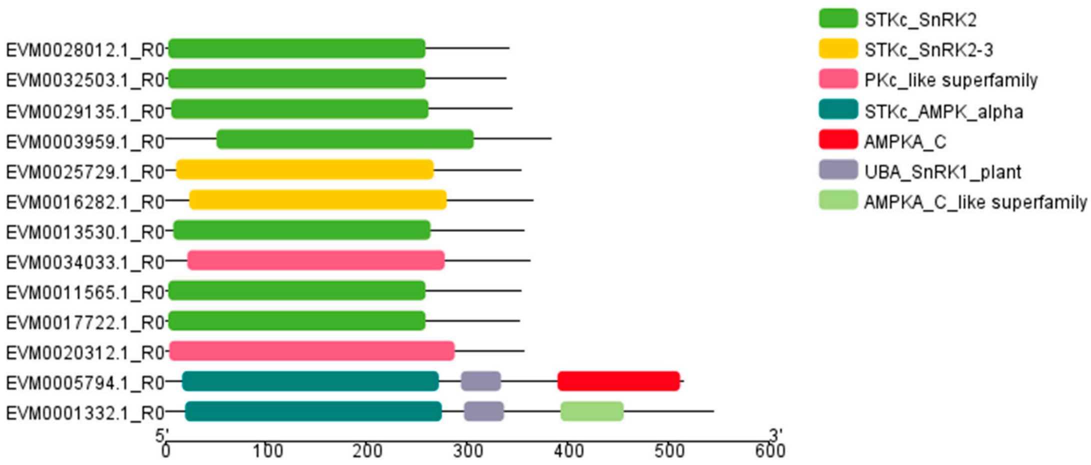

Figure. S3 The trans-membrane structure prediction of AnSnRK2.1.

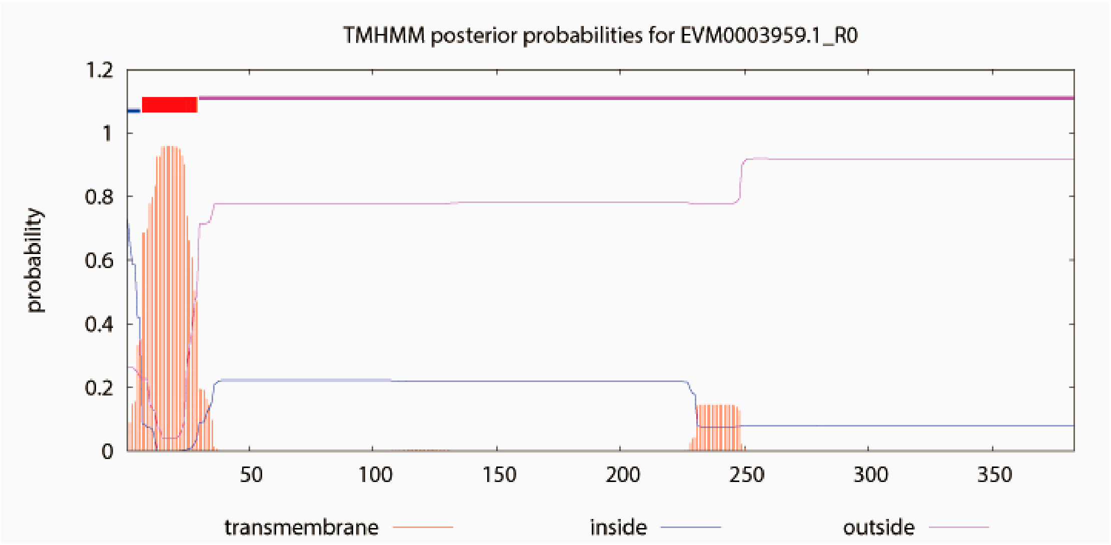

Table S1 SnRK2 genes in *Arabidopsis*, rice, maize, and soybean.

Species	Name	Gene id
Arabidopsis	AtSnRK2.2	At3g50500
	AtSnRK2.3	At5g66880
	AtSnRK2.4	At1g10940
	AtSnRK2.5	At5g63650
	AtSnRK2.6	At4g33950
	AtSnRK2.7	At4g40010
	AtSnRK2.8	At1g78290
	AtSnRK2.9	At2g23030
	AtSnRK2.10	At1g60940
Rice	SAPK1	Os03g27280
	SAPK2	Os07g42940
	SAPK3	Os10g41490
	SAPK4	Os01g64970
	SAPK5	Os04g59450
	SAPK6	Os02g34600
	SAPK7	Os04g35240
	SAPK8	Os03g55600
	SAPK9	Os12g39630
	SAPK10	Os03g41460
Maize	ZmSnRK2.1	AC199054.2
	ZmSnRK2.2	AC186803.4
	ZmSnRK2.3	AC196411.3
	ZmSnRK2.4	AC195225.3
	ZmSnRK2.5	AC190828.2
	ZmSnRK2.6	AC201983.3
	ZmSnRK2.7	AC191130.2
	ZmSnRK2.8	AC206916.1
	ZmSnRK2.9	AC208970.1
	ZmSnRK2.10	AC185635.4
	ZmSnRK2.11	AC196642.4
Soybean	GmSnRK2.1	Glyma01g39020
	GmSnRK2.2	Glyma01g41260
	GmSnRK2.3	Glyma02g15330
	GmSnRK2.4	Glyma02g37090
	GmSnRK2.5	Glyma04g38270
	GmSnRK2.6	Glyma05g31000
	GmSnRK2.7	Glyma05g33170
	GmSnRK2.8	Glyma05g05540
	GmSnRK2.9	Glyma05g09460
	GmSnRK2.10	Glyma06g16780
	GmSnRK2.11	Glyma07g29500

GmSnRK2.12	Glyma07g33120
GmSnRK2.13	Glyma08g14210
GmSnRK2.14	Glyma08g20090
GmSnRK2.15	Glyma08g00770
GmSnRK2.16	Glyma11g04150
GmSnRK2.17	Glyma11g06250
GmSnRK2.18	Glyma12g29130
GmSnRK2.19	Glyma14g35380
GmSnRK2.20	Glyma17g15860
GmSnRK2.21	Glyma17g20610
GmSnRK2.22	Glyma20g01240

Table S2 The primers for qRT-PCR.

Gene name	Primer name	Primer sequence（5'-3')	Product length (bp)
Actin	A-F	TCAGCTGAGCGGGAAATTGT	167
	A-R	GGGCAACGGAATCTTTCAGC	
AnSnRK2.1	1-F	GAAGAGGGTGTTGTTGCTGTTT	96
	1-R	ACCTGGCAACTCCAAAATTCC	
AnSnRK2.2	2-F	GAAGCAAGAGAACCTCCTCCAG	93
	2-R	CACCTCTGCATCCACATCTTCTC	
AnSnRK2.3	3-F	CTTATGCGCCACAAAGAGACA	114
	3-R	ATGGCGAAGACTTCTGTGGT	
AnSnRK2.4	4-F	GTTGAGAATACCATGAACAGCCAG	178
	4-R	CAGTCTCTAGGTCCTCGTCCAT	
AnSnRK2.5	5-F	GGAGCTTACTGAATCAGGTCAAGC	197
	5-R	CTTCCTCTACTTCTGCGTCCAA	
AnSnRK2.6	6-F	GCGGATCATACAAGAAGCAAGG	111
	6-R	CACACAGGTTCCGCATAGTCT	
AnSnRK2.7	7-F	TATCTCCCGAGTGCCGTCAT	159
	7-R	GCTCCTCAAACTGGTTGTTGTTC	
AnSnRK2.8	8-F	GTGAGGACGAGGCAAGGTATT	191
	8-R	AGTAGATTTGGGCTGGGAGTG	
AnSnRK2.9	9-F	GCTCAGCACCACGACTCAA	196
	9-R	GGATAACCGCCAACCAACAT	
AnSnRK2.10	10-F	AGCAAGAAAGCCTCTAAACATCCC	84
	10-R	GCATCAGCATCCAAGTCATCCA	
AnSnRK2.11	11-F	TTCTTAGTGTCCAGTATTCCATTCC	177
	11-R	CCATTATCTTCTCATCCATCAGGTC	
